# Molecular Bases for the Regulation of Adventitious Root Generation in Plants

**DOI:** 10.3389/fpls.2021.614072

**Published:** 2021-01-28

**Authors:** Shi-Weng Li

**Affiliations:** School of Environmental and Municipal Engineering, Lanzhou Jiaotong University, Lanzhou, China

**Keywords:** molecular bases, adventitious rooting, auxin, cytokinin, ethylene, hormone crosstalk

## Abstract

The formation of adventitious roots (ARs) is an ecologically and economically important developmental process in plants. The evolution of AR systems is an important way for plants to cope with various environmental stresses. This review focuses on identified genes that have known to regulate the induction and initiation of ARs and offers an analysis of this process at the molecular level. The critical genes involved in adventitious rooting are the auxin signaling-responsive genes, including the *AUXIN RESPONSE FACTOR* (*ARF*) and the *LATERAL ORGAN BOUNDARIES-DOMAIN* (*LOB*) gene families, and genes associated with auxin transport and homeostasis, the quiescent center (QC) maintenance, and the root apical meristem (RAM) initiation. Several genes involved in cell wall modulation are also known to be involved in the regulation of adventitious rooting. Furthermore, the molecular processes that play roles in the ethylene, cytokinin, and jasmonic acid signaling pathways and their crosstalk modulate the generation of ARs. The crosstalk and interaction among many molecular processes generates complex networks that regulate AR generation.

## Introduction

Root systems are crucial for plant anchor and for the absorption of water and nutrients from the soil. Plant root system consists of primary, lateral, and adventitious roots (ARs) (Osmont et al., 2007). Although the capacity of AR formation is a genetic trait of many plant species (Sorin et al., 2006), in some cases, it can be induced by many environmental stresses, such as flooding, nutrient deficiency, heavy metals, and wounding (Steffens and Rasmussen, 2016). In ecosystems in which soil disturbance is frequent, adventitious rooting can be an alternative or supplement to seed propagation. Thus, AR formation is an important strategy for plant to cope with some environmental stresses (Ramirez-Carvajal and Davis, 2010). In addition, AR formation is an effective method for vegetative propagation of plants and has been successfully used in the production of horticultural species (Li et al., 2009; Steffens and Rasmussen, 2016). The process of AR formation is composed of three successively phases, that is induction, initiation, and expression (De Klerk et al., 1999). The key molecular events occur during root induction and initiation phases. The fate of root competent cells shifts from normal cells to root primordium cells during induction phase, which further develop to form root primordia during initiation phase. A great diverse of molecular and metabolic changes are involved in the induction and initiation of root primordia (Batish et al., 2008). Moreover, the formation of root primordia is regulated by a number of internal and external factors, such as hormones, wounding, and certain environmental stresses (Li et al., 2009; Steffens and Rasmussen, 2016). The physiological and biochemical aspects of AR formation are reasonably well known, however, the molecular processes underlying AR generation, particularly during the early phases, are still largely unknown (Ahkami et al., 2009; Ramirez-Carvajal et al., 2009; Gutierrez et al., 2012; Druege et al., 2016, 2019).

In recent years, there have been increasing interests focused on the molecular bases of adventitious rooting. Many studies have identified a large number of genes directly implicated in the induction and initiation of AR in many plant species (Table 1). These species are the herbaceous dicots *Arabidopsis thaliana* (Sorin et al., 2005, 2006; Gutierrez et al., 2009, 2012), *Solanum lycopersicon* (Negi et al., 2010; Vidoz et al., 2010), *Medicago sativa* (Delphine et al., 2008), *Medicago truncatula* (Nole-Wilson et al., 2005; Imin et al., 2007; Holmes et al., 2008, 2010), *Cucumis sativus* (Xuan et al., 2008; Li et al., 2011), and *Petunia hybrid* (Ahkami et al., 2009) and monocots *Oryza sativa* (Inukai et al., 2005; Liu et al., 2005, 2009, 2011) and *Zea mays* (Taramino et al., 2007), as well as the woody species *Pinus radiata* (Solé et al., 2008; Sozzani et al., 2010; Lucas et al., 2011), *Pinus contorta* (Hemerly et al., 1993; Mews et al., 1996; Lindrotha et al., 2001; Brinker et al., 2004), *Populus trichocarpa* (Rigal et al., 2012), *Populus tremula* × *Populus alba* (Ramirez-Carvajal et al., 2009), and *Malus domestica* (Smolka et al., 2009). However, different types of ARs are formed in these plant species as a normal developmental process or in response to various environmental stresses. Steffens and Rasmussen (2016) proposed a classification of AR subgroups and their definitions. Giving that different AR types are deferentially induced and regulated (Bellini et al., 2014; Pacurar et al., 2014; Druege et al., 2016, 2019; Steffens and Rasmussen, 2016), this review focused on the main four types of ARs, i.e., normal developed-stem roots and etiolation-induced hypocotyl roots in Arabidopsis seedlings; nodal and/or crown roots in rice and maize; *de novo* roots in leaf cultures in alfalfa and leaf explants in Arabidopsis and tobacco, and wounding-induced stem roots in woody cuttings such as *Populus*, *Pinus*, *Malus*, *Eucalyptus*, and *Vitis* plants.

**TABLE 1:** List of genes that have been identified to be involved in AR development.

**Molecular processes**	**Genes**	**Gene descriptions**	**Plant species**	**AR models**	**Functions during AR formation**	**References**
Auxin biosynthesis	*YUCCA1*	flavin monooxygenase	*A. thaliana*	Seedlings	A member of flavin monooxygenase family involved in auxin biosynthesis	Cheng et al., 2006
	*ASA1, ASB1*	*ANTHRANILATE SYNTHASE ALPHA 1, BETA1*	*A. thaliana*	Seedlings	Anthranilate synthase alpha and beta subunits, tryptophan pathway of auxin biosynthesis	Stepanova et al., 2005
	*SUR1, SUR2*	SUPERROOT	*A. thaliana*	Seedlings	Cytochrome P450 Cyp83B1, involved in the indole glucosinolate pathway and regulates endogenous auxin levels	Boerjan et al., 1995; Delarue et al., 1998
Auxin homeostasis	*GH3-3,-5,-6*	GRETCHEN HAGEN3	*A. thaliana, Medicago truncatula*	Seedlings	IAA-amino synthetase, controls auxin homeostasis	Sorin et al., 2005, 2006; Holmes et al., 2010
	*AGO1-3*	ARGONAUTE	*M. truncatulam, A. thaliana*	leaf explants, Seedlings	miRNA-binding proteins, regulate post-transcriptional gene silencing and AR formation	Chen et al., 2009; Morel et al., 2002; Sorin et al., 2006
	*IAR4*	IAA-Alanine Resistant 4	*A. thaliana*	Seedlings	Mitochondrial pyruvate dehydrogenase E1a-subunit, IAA homeostasis	Quint et al., 2009
	*ARRO-1*	Adventitious rooting related oxygenase	*Malus domestica*	Cuttings	2-oxoacid-dependent dioxygenase family protein, involves in hormone homeostasis	Smolka et al., 2009
Auxin receipt	*PagFBL1*	TIR1 homolog	*Populus alba × P. glandulos*a clone 84K	Cuttings	Targets PagIAA28 to regulate AR primordia emergence	Shu et al., 2019
Auxin transport	*PIN1*	PIN-FORMED1	*A. thaliana, Oryza sativa*	Seedlings, nodal roots	Auxin efflux carrier, regulates auxin-dependent AR emergence	Friml et al., 2002; Xu et al., 2005
	*LAX3*	AUXIN1 (AUX1)/LIKE-AUXIN3	*A. thaliana*	Seedlings	Auxin influx carrier	Lee et al., 2019
	*ABCB19*	ATP-binding cassette B19	*A. thaliana*	Root-excised hypocotyls	enhances IAA transport and local IAA accumulation	Sukumar et al., 2013
	*GNOM*	Guanine nucleotide-exchange factor for ADP ribosylation factor	*A. thaliana O. sativa*	Seedlings	Encodes GDP/GTP exchange factor which is required for basal localization of PIN1 protein	Geldner et al., 2004; Blilou et al., 2005
	*CRL2*	Crown rootless2	*O. sativa*	Seedlings	Encodes OsGNOM1	Liu et al., 2009, 2011
	*PP2A*	Protein phosphatase 2A	*A. thaliana*	Stem cuttings	Regulates auxin transport by altering the phosphorylation status of proteins	Ludwig-Muller et al., 2005
	*PID*	PINOID	*A. thaliana*	Seedlings	Encodes a serine/threonine kinase involved in the asymmetrical transport of PINs	Benjamins et al., 2001
	*5NG4*	Nodulin-like	*Pinus taeda*	Cuttings	Transmembrane protein with a possible transport function	Busov et al., 2004
	*CML23-1*	Calmodulin-like	*Populus*	Cuttings	Decreases cytosolic Ca^2+^, regulates AR by IAA level	Xiao et al., 2020
	*WUSa*	WUSCHELa	*Populus tomentosa*	Cuttings	Regulates AR by polar auxin transport	Li J. et al., 2020
Auxin-responsive	*AUX/IAAs*	Auxin/Indole-3-acetic acid	*A. thaliana*	Seedlings	Act as repressors by the interaction with ARFs	Pierre-Jerome et al., 2016
	*IAA33*	Indole-3-acetic acid inducible 33	*M. truncatula*	Leaf cultures	Root meristem formation	Holmes et al., 2008, 2010
	*IAA6, IAA9, IAA17*		*A. thaliana*	Seedlings	Interact with ARF6 and/or ARF8	Li et al., 2016, 2017; Lakehal et al., 2019a
	*ARF6, ARF7, ARF8, ARF10, ARF17, ARF19*	Auxin response factors	*A. thaliana*	Seedlings	Regulate the expression of auxin response genes. Interact with Aux/IAA to regulate AR	Sorin et al., 2005, 2006; Li et al., 2006; Gutierrez et al., 2009, 2012; Bellini et al., 2014; Li et al., 2016, 2017; Lee et al., 2019
	*SAUR15*	*SMALL AUXIN-UP RNA*	*A. thaliana*	Seedlings	Acts downstream of ARFs to promote IAA synthesis and release the activity of membrane H^+^-ATPases	Yin et al., 2020; Copeland, 2020
Meristem and primordium formation	*LBD15/ASL1*	Lateral organ boundaries domain (LOB domain)/ Asymmetric leaves 2-like	*M. truncatula*	Leaf cultures	Regulates root meristem formation	Holmes et al., 2010
	*LBD16,LBD29*	LOB domain	*A. thaliana*	Seedlings	Downstream genes of ARF7 and ARF19, regulates cell cycle in response to auxin	Lee et al., 2019; Welander et al., 2014
	*LBD18/ASL20*	LOB domain	*A. thaliana, M. truncatula*	Seedlings, leaf cultures	Downstream genes of ARF7 and ARF19	Holmes et al., 2010; Lee et al., 2019
	*RTCS, RTCL*	Rootless concerning crown and seminal roots, RTCS-Like	*Zea mays*	Shoot-borne roots	LOB domain proteins, regulate shootborne root primordia	Taramino et al., 2007
	*ARL1*	Adventitious rootless 1	*O. sativa*	Seedlings	LOB domain auxin-responsive factor, cell dedifferentiation	Liu et al., 2005
	*ARL2*	Adventitious rootless 2	*O. sativa*	Seedlings	Involves in ethylene signaling, affects AR formation	Liu et al., 2011
	*SPL2,10,26*	SQUAMOSA promoter binding protein-like	*A. thaliana*	Seedlings	Binds to the promoters of AP2/ERFs resulting in attenuation of root induction by reducing auxin accumulation	Gandikota et al., 2007; Ye et al., 2020a
	*CRL1/ARL1*	Crown rootless1/Adventitious rootless 1	*O. sativa*	Seedlings	ASYMMETRIC LEAVES2 (AS2)/LOB domain proteins, regulates crown root formation	Inukai et al., 2005
	*LRP1*	Lateral root primordium 1	*A. thaliana, M. truncatula*	Seedlings, leaf cultures	Zinc finger transcription factor, regulates early stages of AR development	Smith and Fedoroff, 1995; Holmes et al., 2010
	*bZIP53*		*Populus*	Cuttings	Regulates AR via expression of *IAA4-1/2*	Zhang et al., 2020
	*RML1*	Root meristemless1/Cadmium sensitive 2	*A. thaliana, M. truncatula*	Seedlings, leaf cultures	gamma-glutamylcysteine synthetase, involved in initiation and maintenance of cell division	Vernoux et al., 2000b; Holmes et al., 2010
	*AIL PtAIL1, 5*,	AINTEGUMENTA-like	*A. thaliana, P. trichocarpa*	Seedlings, Cuttings	AP2/ERF family, maintains meristematic or division-competent states	Nole-Wilson et al., 2005; Rigal et al., 2012; Trupiano et al., 2013
	*PLT1,2 PtPLT1.1,1.2*	PLETHORA	*A. thaliana, M. truncatula M. truncatula, Populus*	Seedlings, leaf cultures, cuttings	AP2/EREBP subfamily, promotes stem cell identity and maintenance, mitotic activity, and cell differentiation	Nole-Wilson et al., 2005; Galinha et al., 2007; Imin et al., 2007; Holmes et al., 2010; Rigal et al., 2012
	*ANT*	AINTEGUMENTA	*A. thaliana*	Seedlings	Establishment and maintenance of meristems	Nole-Wilson et al., 2005
	*BBM, PtBBM2*	BABY BOOM	*M. truncatula Populus*	Leaf cultures, cuttings	Establishment and maintenance of meristems	Nole-Wilson et al., 2005; Imin et al., 2007; Holmes et al., 2010; Trupiano et al., 2013
	*RRD1,2,4*	Root redifferentiation 1,2,4	*A. thaliana*	Seedlings	Active cell proliferation and competence for cell proliferation	Konishi and Sugiyama, 2006
	*RPD1*	Root primordium defective 1	*A. thaliana*	Seedlings	Active cell proliferation during root primordium development	Konishi and Sugiyama, 2006
	*RID2,5*	Root initiation defective 2,5	*A. thaliana*	Seedlings	Nuclear methyltransferase-like protein, Involves in pre-rRNA processing, stimulates cell proliferation to form ARs	Konishi and Sugiyama, 2003; Ohbayashi et al., 2011
	*MOR1*	Microtubule organization	*A. thaliana*	Seedlings	Microtubule-associated protein, root meristem initiation	Konishi and Sugiyama, 2003
	*RGD1-3*	Root growth defective	*A. thaliana*	Seedlings	Regulate cell proliferation during dedifferentiation of cells	Sugiyama, 2003
	*RCH1,2*	Root clavata1-homologue	*M. truncatulam*	Leaf cultures	Leucine rich repeat receptor-like kinase, specific to the transition zone of root initiation	Holmes et al., 2010
	*HBT*	HOBBIT	*A. thaliana*	Seedlings	Root meristem formation	Willemsen et al., 1998
	*CAND1*	Cullin-associated and neddylation-dissociated 1	*O. sativa*	Crown roots	Play roles in the G2/M cell cycle transition during the emergence of crown root	Wang et al., 2011
	*SHR*	SHORT-ROOT	*Pinus radiata, A. thaliana*	Cuttings, seedlings	GRAS family, regulates SCR expression and cell cycle component CYCD6;1, is required for root primordia of ARs	Solé et al., 2008; Sozzani et al., 2010; Lucas et al., 2011
	*SCR*	SCARECROW	*P. Radiate, A. thaliana*	Cuttings, seedlings	GRAS family, similar to SHR, controls cell division	Sozzani et al., 2010; Legue et al., 2014
	*SCL, SCL1*	SCARECRO W-like 1	*P. radiata, Castanea sativa, Juglans nigra*	Cuttings	Auxin-signaling pathway, plays a role during the earliest stages of AR formation	Sanchez et al., 2007; Vielba et al., 2011; Stevens et al., 2017
	*CDC2*	PSTAIRE CDC2 cyclin-dependent kinase	*A. thaliana, P. contorta*	Seedlings, cuttings	Involves in a developmental switch between mitotic cell division and post-mitotic cell differentiation and maintains proliferation competence	Hemerly et al., 1993; Mews et al., 1996; Lindrotha et al., 2001
	*PINHEAD/ZWILLE-Like*	PINHEAD/ZWILLE-Like	*P. contorta*	Cuttings	Regulates root meristem formation	Brinker et al., 2004
	*WOX4*	WUSCHEL related homeobox LkWOX4	*Larix kaempferi*	Cuttings	Regulates root apical meristem formation	Wang et al., 2020
	*WOX5*	WUSCHEL related homeobox	*M. truncatula, Populus*	Leaf cultures, cuttings	Regulates root apical meristem formation	Imin et al., 2007; Holmes et al., 2010; Li et al., 2018
	*WOX5,7*	WUSCHEL related homeobox	*A. thaliana*	Seedlings	Downstream targets of WOX11/12	Hu and Xu, 2016
	*WOX11,12*	WUSCHEL related homeobox	*A. thaliana, O. sativa, Populus*	leaf explants, crown root, cuttings	Enhance LBD16 and 29, resulting in the first-step cell fate transition from a leaf procambium to a root founder cell	Liu et al., 2014; Zhao et al., 2009; Xu et al., 2015
	*ROLB*	Rooting-locus B	*N. tabacum*	Leaf explants	Interacts with 14-3-3 protein to alter developmental plasticity and regulates AR formation	Moriuchi et al., 2004
	*NAC1*	petunia NAM and Arabidopsis ATAF1, ATAF2, and CUC2	*A. thaliana*	Leaf explants	Affects the emergence of AR tips via upregulating CEPs.	Chen et al., 2016
	*ROLD*	Rooting-locus D	*A. thaliana*	seedlings	Regulates AR meristem formation	Falasca et al., 2010
miRNAs	*miR156*		*Solanum lycopersicum, Nicotiana tabacum, Malus xiaojinensis, A. thaliana*	Seedlings, cuttings, leaf explants	Targets and represses a group of SPLs, promotes AR formation	Zhang et al., 2011; Feng et al., 2016; Xu et al., 2017; Ye et al., 2020b
	*miR160a*	peu-miR160a	*Populus*	Cuttings	Regulates *PeARF17.1*/*PeARF17.2*	Liu et al., 2020
	*miR167a*		*Populus*	Cuttings	Regulates *Pe ARF 6s, 8s*	Cai et al., 2019
	*miRNAs*		*Malus domestica*	Cuttings	Involves in AR formation	Li et al., 2019
GA pathway	*PtHDT902*	Histone deacetylase	*Populus trichocarpa*	Cuttings	Represses AR by increasing GA biosyntheses genes	Ma et al., 2020
NO signaling	*NIR*	nitrate reductase	*Eucalyptus grandis*	Cuttings	Nitric oxide production	Abu-Abied et al., 2012; Li S.W. et al., 2020
Secondary pathway	*CHS*	Chalcone synthase	*Juglans nigra*	Cuttings	Key enzyme in flavonoid biosynthesis, negatively respond to auxin	Stevens et al., 2017
	*HO1*	Heme oxygenase	*Cucumis sativus*	Root-excised seedlings	Catalyzes the degradation of heme	Li et al., 2011; Xuan et al., 2008
Cell wall modification	*CEP1,2*	KDEL-tailed Cys endopeptidase	*A. thaliana*	Leaf explants	Degrades extensin proteins in the cell wall, promotes emergence of regenerated root tips	Chen et al., 2016
	*EXT1*	Extensin	*Vitis vinifera, A. thaliana*	Cuttings, seedlings	Strengthens cell walls during wound healing	Thomas et al., 2003; Merkouropoulos and Shirsat, 2003
	*PRP1, 2*	Proline-rich protein 1,2	*Vitis vinifera*	Cuttings	Proline-rich proteins, cell wall modification	Thomas et al., 2003
	*RHD3*	ROOT HAIR DEFECTIVE	*A. thaliana, Populus*	Seedlings, cuttings	GTP-binding protein, regulate cell wall biosynthesis and actin organization	Hu et al., 2003; Xu et al., 2012

All the genes that function in auxin perception, transport, and homeostasis, as well as in cell division, cell wall synthesis, cell wall weakening, root meristem formation, and quiescent center (QC) maintenance have been demonstrated to modulate AR formation (Brinker et al., 2004; Sorin et al., 2006; Holmes et al., 2010). Transcription factors (TFs), such as the APETALA2/ETHYLENE RESPONSIVE ELEMENT BINDING (AP2/ERF), MYB, NAC, WRKY, and bHLH families, have been found to mediate AR formation (Rigal et al., 2012; Druege et al., 2016, 2019). The previous studies have shown that, at the molecular level, complex networks are implicated in the regulation of AR generation in plants (Table 1).

## Molecular Bases Associated With Auxin Signaling During Adventitious Rooting

Auxin has been well known to act as a central regulator controlling adventitious rooting in plants. Increasing evidences have shown that auxin signaling-related genes are the most important molecular bases for the initiation of AR in plants. Three kinds of auxin signaling genes are involved in the AR process, i.e., auxin synthesis- and homeostasis-related genes, auxin transport-related genes, and auxin-responsive genes.

### Auxin Synthesis-, Homeostasis-, and Receptor-Related Genes Mediate AR Formation

**FIGURE 1:**
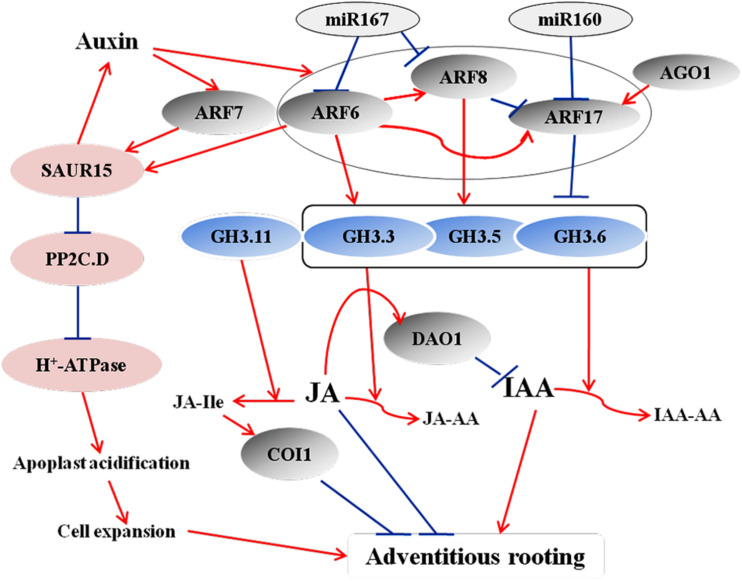
A model illustrating how ARFs, miRNAs, and GH3s proteins regulate adventitious rooting by controlling IAA and JA homeostasis during AR formation in Arabidopsis seedlings. Auxin controls adventitious root initiation by activating ARF6 and ARF8, leading to the downregulation of inhibitory COI1 signaling (Gutierrez et al., 2009, 2012). ARF6, ARF8, and ARF17 and their regulatory miRNAs interact in a complex network and act upstream of *GH3.3*, *GH3.5*, and *GH3.6*. ARF6 and ARF8 are positive regulators of these three *GH3* genes, whereas ARF17 is a negative regulator. The three GH3 proteins control free IAA levels and JA homeostasis. The JA level determines the JA-Ile level. JA-Ile negatively regulates adventitious rooting by activating the COI1 signaling pathway. AGO1 upregulates *GH3* genes by downregulating *ARF17*, thereby regulating adventitious rooting through this pathway, as well. Another feedback circuit consist of DAO1, IAA, and COI1-dependent JA signaling. COI1-dependent JA signaling actives the expression of DAO1, which in turn regulates IAA homeostasis by irreversible degradation (Lakehal et al., 2019b). Copeland (2020) and Yin et al. (2020) proposed a model of the involvement of SAUR15 in auxin signaling-mediated AR formation in Arabidopsis seedlings. ARFs directly bind to the promoter of *SAUR15* to activate its expression. SAUR15 promote AR formation via two pathways. SAUR15 inhibit PP2C.D activity, leading to the release of membrane H^+^-ATPase activity, which then causes apoplastic acidification and cell expansion, thereby facilitating AR emergence. SAUR15 also activates auxin biosynthesis to promote AR formation. Red arrows represent positive regulation, and blue bars represent negative regulation.

The genes involved in IAA synthesis and homeostasis have been demonstrated to directly influence AR formation. *YUCCA* encodes flavin monooxygenase that is involved in tryptophan-dependent indole-3-acetic acid (IAA) biosynthesis (Cheng et al., 2006; Mashiguchi et al., 2011). The overexpression of *YUCCA6* in Arabidopsis causes typical auxin overproduction phenotypes and increases free IAA to maintain local IAA levels in the root apical meristem (Rovere et al., 2013). Studies in Arabidopsis and rice showed that *YUCCA6* directly mediate AR formation by increasing IAA levels not only via direct biosynthesis but also by affecting the expression of *GRETCHEN HAGEN3* (*GH3*) family genes (Kim et al., 2007; Yamamoto et al., 2007). The *GH3* family genes encode IAA-amido synthetases, which catalyze the conjugation of various amino acids to auxin and jasmonate, and consequently control the free IAA level (Ljung et al., 2002; Staswick et al., 2005; Sorin et al., 2006; Ludwig-Muller et al., 2009). The member *GH3-3* was shown to be involved in the activation of AR initiation in both the hypocotyls and stems of *Arabidopsis* (Welander et al., 2014). *WEAK ETHYLENE INSENSITIVE2/ANTHRANILATE SYNTHASE alpha 1* (*WEI2*/*ASA1*), *WEAK ETHYLENE INSENSITIVE7/ANTHRANILATE SYNTHASE beta 1* (*WEI7/ASB1*), and *TRYPTOPHAN SYNTHASE BETA 1* (*TSB1*) encode α- and β-subunits of anthranilate synthase, which is a rate-limiting enzyme of Trp-dependent IAA biosynthesis; disruption of their expression reduces auxin biosynthesis and results in a reduction in AR number in Arabidopsis (Stepanova et al., 2005). *SUPERROOT1* (*SUR1*) encodes a C-S lyase that converts S-alkyl-thiohydroximate into thiohydroximate, which is the first step of indole glucosinolate biosynthesis. *SUR2* encodes cytochrome P450-dependent monooxygenase CYP83B1, which blocks the synthesis of indole glucosinolates. The expression of these two genes increases endogenous IAA levels and thereby promoting adventitious rooting (Boerjan et al., 1995; Delarue et al., 1998; Mikkelsen et al., 2004). *ARGONAUTE1* (*AGO1*) encodes a miRNA-binding protein that regulates post-transcriptional gene silencing (Morel et al., 2002; Vaucheret et al., 2004). De Almeida et al. (2010) demonstrated that AGO1 controls the genes related to auxin homeostasis and AR development in *Eucalyptus globules*. Studies in the *ago1* mutants of Arabidopsis reveal that AGO1 alter auxin homeostasis by upregulating the expression of the auxin response factor *ARF17* and GH3-like genes, leading to the defect in adventitious rooting (Sorin et al., 2005, 2006; Figure 1). *IAA ALANINE RESISTANT4* (*IAR4*) encodes a putative mitochondrial pyruvate dehydrogenase E1α subunit that is involved in IAA metabolism and homeostasis. In *Arabidopsis*, this enzyme catalyzes the conversion of indole-3-pyruvate to IAA-CoA (Quint et al., 2009), resulting in low free IAA and AR formation. In *Malus domestica, ADVENTITIOUS ROOTING RELATED OXYGENASE* (*ARRO-1*) encodes 2-oxoacid-dependent dioxygenase, which can oxidize IAA leading to the reduction of AR initiation (Smolka et al., 2009). Calmodulin is involved in AR formation by modulating IAA content. A recent study showed that the poplar transgenic lines with overexpressed the calmodulin-like protein (CML) family gene, *PdeCML23-1*, exhibited more ARs and higher IAA accumulation in cuttings (Xiao et al., 2020).

Auxin receptor genes have also been demonstrated to be involved in AR formation. A recent study identified a homolog gene of Arabidopsis auxin receptor TIR1, *PagFBL1*, in poplar, which highly expressed in the stem cambium and secondary phloem during early phase of AR formation in cuttings. The transgenic cuttings with overexpression of *PagFBL1* displayed higher ARs than the wild type, while knock-down of *PagFBL1* reduced ARs. Furthermore, PagFBL1 interacts with PagIAA28.1 and PagIAA28 to release ARFs (Shu et al., 2019).

### The Interactions Among ARFs, GH3s, miRNAs, and SPLs During AR Formation

**FIGURE 2:**
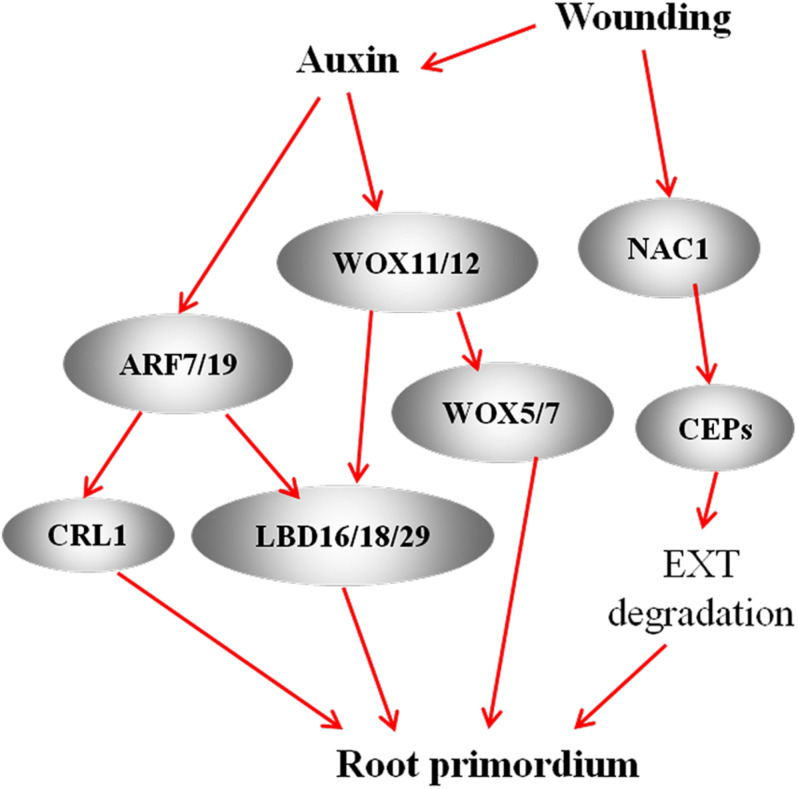
A model of interaction among WOXs, LBDs, and ARFs regulates the formation of AR primordia during AR formation. In the AR model of Arabidopsis seedlings, ARF7/19 activate *LBD16/18/29* expression to promote AR formation (Lee et al., 2019). In the rice model, ARFs target CRL1 to regulate AR (Inukai et al., 2005). During *de novo* AR formation in Arabidopsis leaf explants, wounding-induced auxin activate the expression of *WOX11* and *WOX12*, which upregulate *LBD16* and *LBD29* (Liu et al., 2014) and directly activate *WOX5* and *WOX7* to promote AR (Hu and Xu, 2016). In *de novo* AR model in leaf explant of Arabidopsis, wounding-induced the expression of *NAC1* highly upregulated the expression of Cys endopeptidase-coding gene *CEP1* and *CEP2*. CEP plays a role in degradation of extensin (EXT) in the cell wall and thus conduces to the emergence of AR primordia (Chen et al., 2016). Red arrows represent positive regulation.

**FIGURE 3:**
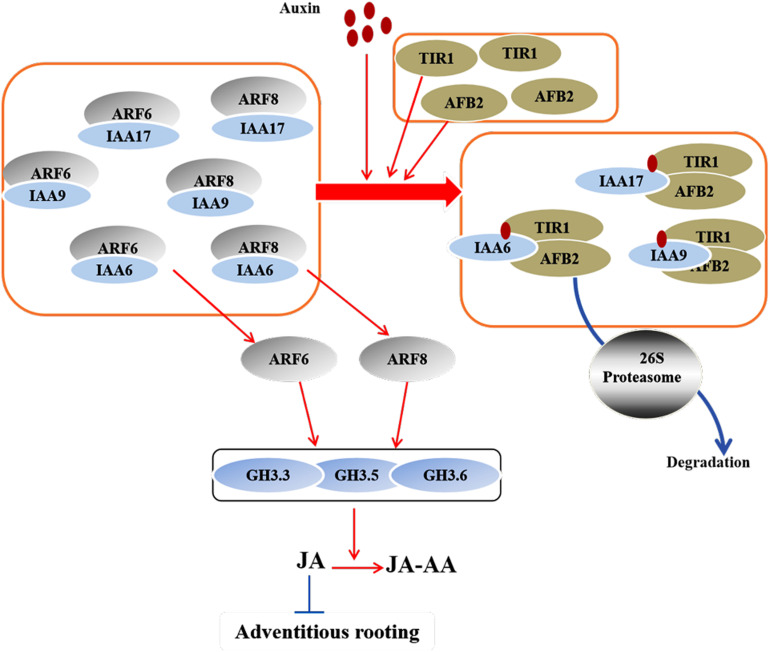
A model that was recently proposed by Lakehal et al. (2019a) illustrates the interaction between IAAs and ARFs during AR formation in Arabidopsis hypocotyls. The AUX/IAA family proteins IAA6, IAA9, and IAA17 interact with ARF6 and ARF8 to repress their activity at low IAA level. At high IAA level, the Aux/IAA proteins form an auxin coreceptor complex with TIR1 and AFB2 and are degraded through the 26S proteasome. The released ARF6 and ARF8 further initiate the expression of auxin-responsive genes for AR formation. In this model, IAA6, IAA9, and IAA17 repress the expression of *GH3.3, GH3.5*, and *GH3.6*, while ARF6 and ARF8 active their expression. Red arrows represent positive regulation, and blue bars represent negative regulation.

The ARF TFs control the expression of auxin-responsive genes at the transcriptional level and regulate plant development through auxin signaling (Guilfoyle and Hagen, 2007; Paponov et al., 2008; Bargmann et al., 2013; Chaiwanon and Wang, 2015). In Arabidopsis, ARF6, ARF8, and ARF17 control the expression of *GH3.3*, *GH3.5*, and *GH3.6*, and the upregulation of *GH3s* promote AR formation (Sorin et al., 2006; Gutierrez et al., 2012; Figure 1). ARF17 negatively regulates adventitious rooting by repressing the expression of *GH3* genes, thereby altering auxin homeostasis; in contrast, ARF6 and ARF8 positively regulate adventitious rooting by inducing the expression of *GH3* genes (Sorin et al., 2005, 2006; Gutierrez et al., 2009, 2012; Lakehal et al., 2019a). Another study proposed that the activation of *GH3* genes might be due to the auxin-induced degradation of ARF6 and ARF8 (Pacurar et al., 2014). Furthermore, ARF6, ARF8, and ARF17 interact at the transcriptional level (Gutierrez et al., 2009). At the post-transcriptional level, *miR160* and *miR167* regulate the expression of *ARF6*, *ARF8*, and *ARF17*. ARF6, ARF8, and ARF17, in turn, affect the expression of miR160 and miR167 (Gutierrez et al., 2012). These results suggest that a subtle balance between the repressor ARF17 and the activators ARF6 and ARF8 control the initiation of ARs via a complex regulatory network (Sorin et al., 2005; Gutierrez et al., 2009; Figure 1). Recently, Lee et al. (2019) identified a novel auxin signaling module controlling AR formation in Arabidopsis hypocotyls, in which ARF7 and ARF19 act as positive regulators via activating the downstream members LBD16 and LBD18 (Figure 2). Cai et al. (2019) showed that, in poplar, the cuttings with overexpression of *PeARF8.1* gene displayed more ARs than the wild type, and miR167a targets *PeARF6s* and *PeARF8s* to regulate AR formation. Similarly, Liu et al. (2020) showed that the poplar peu-miR160a negatively regulated the genes *PeARF10.1*, *PeARF16.1*, *PeARF16.2*, *PeARF16.3*, *PeARF17.1*, and *PeARF17.2*. Overexpressing *PeARF17.1* or *mPeARF17.2* significantly increased AR number in the cuttings. In this network, miRNAs function as modulators to fine-tune adventitious rooting (Gutierrez et al., 2009). A recent study by Lakehal et al. (2019a) showed that *AUX/IAA* family genes are also involved in AR formation in Arabidopsis hypocotyls, of which *IAA6, IAA9*, and *IAA17* repress ARF6 and ARF8 by interacting with them. Furthermore, *IAA6, IAA9*, and *IAA17* repress the expression of *GH3.3, GH3.5*, and *GH3.6* during AR formation (Figure 3).

The stimulative role of miR156 in *de novo* root formation has been proved in Arabidopsis (Aung et al., 2017; Ye et al., 2020b), *Malus xiaojinensis* (Xu et al., 2017), and poplar (Cai et al., 2019). In Arabidopsis, miR156 repress its targets SQUAMOSA PROMOTER BINDING PROTEIN-LIKE (SPL) TFs to maintain juvenile traits. The member SPL10 inhibits AR regeneration by reducing wounding-induced auxin burst in the juvenile leaf explants. In the adult leaf explants, the members SPL2/10/11 bind to the promoters of AP2/ERFs resulting in attenuation of AR induction by reducing auxin level (Gandikota et al., 2007; Ye et al., 2020a). In the *M. xiaojinensis* leaf explants, the high expression of miR156 is required for auxin-induced AR formation, and *MxSPL26* is involved in this process (Xu et al., 2017). In the cuttings of *E. grandis*, *EgSPL2* and *EgSPL5* negatively affect AR formation (Abu-Abied et al., 2012). These results suggest that miR156-SPLs network control AR formation.

Recently, Yin et al. (2020) revealed the interaction between *SMALL AUXIN-UP RNA15* (*SAUR15*) and ARFs in controlling AR formation. ARF6 and ARF7 directly bind to the promoter of *SAUR15* to promote its expression. *SAUR*15 promote AR formation via enhancing auxin accumulation and inhibiting the activity of PP2C-D type 2C protein phosphatase, which activates the activity of membrane H^+^-ATPases leading to apoplastic acidification, thereby facilitating cell expansion and AR emergence (Copeland, 2020; Figure 1).

### Changes in Expression of Auxin Transporter-Related Genes During AR Formation

Polar auxin transport (PAT) results in the differential distribution of auxin in certain tissues, which directly triggers the initiation of ARs (Ludwig-Muller et al., 2005). Studies in *Arabidopsis* hypocotyls showed that auxin moves out of the shoot apex and accumulates in the vascular parenchyma adjacent to pericycle founder cells, causing an IAA gradient that is essential for adventitious rooting (Rovere et al., 2013; Sukumar et al., 2013). Three main classes of auxin transporters determine the differential distribution of auxin in the tissues, including the influx carriers AUXIN1/LIKE AUX1 (AUX1/LAX) and two kinds of efflux carriers, i.e., the ATP-BINDING CASSETTE subfamily B (ABCB) and the PIN- FORMED (PIN) proteins. Arabidopsis has four highly conserved AUX1 family multigenes (i.e., *AUX1* and *LIKE AUX1* [*LAX*] genes *LAX1*, *LAX2*, and *LAX3*), eight PIN family members, and 29 *ABCB* members, which provide roust functional redundancy in regulating auxin transport and homeostasis (Guan et al., 2019).

PIN1 mediates AR induction by facilitating the lateral auxin efflux from the vascular system to the pericycle founder cells (Péret et al., 2012). The auxin induced *PIN1* expression is essential for AR formation in cotyledon cuttings of *Mangifera indica* (Li et al., 2012) and auxin-dependent AR emergence in rice (Xu et al., 2005). *PIN* expression reinforces auxin gradients, further regulating the expression of meristem cell-specific genes *PLETHORA1* (*PLT1*) and *PLT2* (Benkova et al., 2003; Aida et al., 2004; Blilou et al., 2005). The PIN-LIKES (PILS) family proteins are novel putative auxin transport facilitators localized to the endoplasmic reticulum and participate in the regulation of intracellular IAA transport and auxin availability (Barbez et al., 2012). Recent studies examined the expression profile of PIN family members in olive and tomato cuttings. During IBA-induced AR formation in olive cuttings, *OePIN2b* and *OePIN5b* were highly upregulated, whereas, *OePIN1a-c*, *OePIN3a-c*, *OePIN6*, and *OePIN8* were downregulated (Velada et al., 2020). In tomato cuttings, *SlPIN*2, *SlPIN* 3, *SlPIN* 4, and *SlPIN* 7 were involved in AR induction and initiation (Guan et al., 2019).

Auxin regulates *PIN* expression through the ARF family proteins (Vieten et al., 2005) and increases PIN levels and activity at the plasma membrane (Paciorek et al., 2005). Several proteins are involved in controlling the asymmetrical transport of PINs and polar auxin transport in *Arabidopsis* and rice (Geldner et al., 2003, 2004; Morita and Kyozuka, 2007), such as a Ser/Thr kinase encoded by *PINOID* (*PID*) (Benjamins et al., 2001) and a large guanine nucleotide exchange factor for ADP-ribosylation factor (GNOM), which is a small GTPase. The rice OsGNOM1 encoded by *CRL2*, can affect polar auxin transport by disturbing the vesicle trafficking of PIN1, thereby affecting the formation of AR primordia (Kitomi et al., 2008; Liu et al., 2009, 2011).

The auxin influx carrier AUX1 is also localized asymmetrically and takes up auxin in a pH-dependent, high-affinity manner in the root meristem cells (Blilou et al., 2005; Yang et al., 2006). Studies have shown that the genes *AUX1* and *LIKE-AUX1-3* (*LAX3*) in *Arabidopsis* (Péret et al., 2012; Rovere et al., 2016; Lee et al., 2019), *AUX3* and *AUX4* in *Mangifera indica* (Li et al., 2012), and *SlLAX1* in tomato (Pattison and Catala, 2012) are essential for AR formation.

As auxin efflux carriers, ABCBs act in concert with the PIN proteins (Blakeslee et al., 2007; Mravec et al., 2008). Four *Arabidopsis* mutants, *pin1, pin3, pin7*, and *abcb19*, display significant reductions in AR formation, indicating that PIN and ABCB proteins mediate AR formation through affecting auxin efflux. Furthermore, the overexpression of *ABCB19* increases auxin transport and IAA accumulation, driving the initiation of ARs in the hypocotyl of *Arabidopsis*, whereas suppression of *ABCB19* led to a reduction in auxin level in competent cells, and resulted in less ARs (Christie et al., 2011; Sukumar et al., 2013). In *Pinus taeda, 5NG4* encodes an auxin-induced transmembrane protein which probably acts as auxin transporter and is involved in AR formation in hypocotyl cuttings (Busov et al., 2004).

Besides, some proteins mediate AR formation by affecting the status of auxin transporters. For example, the *Arabidopsis* gene *PP2A* encodes a type 2A Ser/Thr protein phosphatase, which is shown to modulate AR formation by modifying the phosphorylation status of auxin transporters and affecting polar auxin transport (Ludwig-Muller et al., 2005).

## Auxin-Responsive LOB-Domain TFs Are Involved in Adventitious Rooting

The plant-specific LATERAL ORGAN BOUNDARIES-DOMAIN (LOB-domain, LBD) TFs have been shown to modulate AR formation (Hochholdinger and Zimmermann, 2008) through regulating cell division and cell wall modification during AR generation. These *LBD* family members include the rice *ADVENTITIOUS ROOTLESS1* (*ARL1*) (Liu et al., 2005) and *CROWN ROOTLESS1* (*CRL1*), which encodes a member of AS2/LOB protein family (Inukai et al., 2005), the maize *ROOTLESS CONCERNING CROWN AND SEMINAL ROOTS* (*RTCS*) (Taramino et al., 2007), the *Medicago MtLOB29* (Holmes et al., 2010), and the *Arabidopsis LBD16*, *LBD18* and *LBD29* (Shuai et al., 2002; Lee et al., 2019). ARL1 and CRL1 positively regulate the initiation of AR primordia in an auxin-dependent manner in rice (Inukai et al., 2005; Liu et al., 2005; Kitomi et al., 2008). *RTCS* and *RTCL* (*RTCS-LIKE*) regulate the initiation and maintenance of root primordia. The auxin-responsive RTCS inhibits AUXIN BINDING PROTEIN1 (ABP1), while activates calmodulin resulting in the activation of downstream calcium-dependent signaling (Taramino et al., 2007). Members of the LOB/ASYMMETRIC LEAVES2 (ASL2) TF family are expressed in response to auxin and are induced during AR generation in *Medicago* leaf cultures (Holmes et al., 2010). In *Arabidopsis*, auxin induces the expression of *LBD16*, *LBD18*, and *LBD29* in hypocotyls and stems leading to AR formation; LBD16 plays a direct role in root initiation, while LBD29 acts an indirect regulator of root differentiation and development (Welander et al., 2014). Moreover, the rice CRL1 and Arabidopsis *LBD16* and *LBD18* are also directly promoted by ARF7 and ARF19 via binding to their promoters (Inukai et al., 2005; Lee et al., 2019; Figure 2).

## Genes That Are Involved in AR Primordium Formation Play Regulative Roles in Quiescent Center (QC) Maintenance and Root Apical Meristem (RAM) Initiation

The initiation of RAM and maintenance of QC are key processes for AR induction. Two steps are required for cell fate transition to RAM formation. Firstly, regeneration-competent cells dedifferentiate to become root founder cells. Secondly, the root founder cells transform into root primordium cells and initiate mitosis to form RAM (Hu and Xu, 2016). The most prominent molecular processes occur during the QC and RAM formation. Many genes and several TF family genes have been identified to be involved in the regulation of QC and RAM initiation.

### GRAS Family TFs

The *SHORTROOT* (*SHR*) and *SCARECROW* (SCR) genes encode closely related TFs belonging to the *GRAS* (*GAI*, *RGA*, and *SCR*-like) gene family, and their products play a role in establishing and maintaining the RAM (Aida et al., 2004; Xu et al., 2006; Sanchez et al., 2007; Solé et al., 2008). SCR controls cell division and differentiation leading to the formation of AR primordium in *Arabidopsis* (Heidstra et al., 2004; Wildwater et al., 2005; Legue et al., 2014). The *SCARECROW-LIKE* (*SCL*) family genes, such as *PrSCL1* from *Pinus radiata* and *CsSCL1* from *Castanea sativa* (Sanchez et al., 2007; Solé et al., 2008; Vielba et al., 2011), *PrSHR* from *Populus radiata* (Solé et al., 2008), and *SCR* from *Populus trichocarpa* (Rigal et al., 2012), are activated during the earliest stages of AR formation in the cuttings. In *Arabidopsis*, SCR interacts with SHR to activate downstream target genes, thereby regulating RAM and QC (Vernoux and Benfey, 2005) and the positioning of the stem cell niche (Lucas et al., 2011). SHR regulates the expression of direct target genes, including the D-type cyclin gene *CYCD6;1* and the cyclin-dependent kinase genes *CDKB2;1* and *CDK2;2* (Sozzani et al., 2010), and induces endodermal cell identity and the expression of *SCR*. SCR controls asymmetrical cell divisions and limits the movement of SHR (Cui et al., 2007). These results indicate that SCR/SHR complex activates D-type CYCLIN genes and involves in initiation of cell dedifferentiation in Arabidopsis (Xu et al., 2006). Furthermore, a study found that the *shr* mutant exhibits a loss of PIN protein, indicating that SHR also affects PIN protein abundance (Lucas et al., 2011). Using a laser capture microdissection protocol for site-specific RNA isolation and analysis, Stevens et al. (2017) examined gene expression changes during AR formation in black walnut cuttings. The results indicated that, in rooting-competent cuttings, root primordium cells exhibited the greatest transcript abundance. In juvenile rooting-competent cells, the *SCL* expression displayed 23- to 24-fold increase, *ARF6* and *ARF8* as well as *SHR* expression displayed 2- to 4-fold increase, implying the importance of these genes for root primordium generation.

### AP2/ERPB2 Family TFs

The five *AINTEGUMENTA*-like (*AIL*) family of AP2/ERPB2 domain TFs genes, including *PLETHORA1* (*PLT1*), *PLT2*, *AINTEGUMENTA* (*ANT*), *AINTEGUMENTA-Like* (*AIL*), and *BABY BOOM* (*BBM1*), are expressed in all dividing tissues and are required for AR primordium formation (Xu et al., 2006; Galinha et al., 2007; Passarinho et al., 2008). In Arabidopsis, high expression of *PLT1* and *PLT2* was detected in the root meristem, which are required for QC identity and the patterning of the root stem cell niche within the RAM (Galinha et al., 2007; Imin et al., 2007; Holmes et al., 2008). PLT increases *PIN* expression leading to the flux of auxin into the RAM (Blilou et al., 2005). In *Populus trichocarpa*, the expression of *PtPLT1.1* and *PtBBM* increase greatly during the organization and differentiation of AR primordia in the cuttings (Rigal et al., 2012). *BBM* encodes an embryo-expressed TF that is involved in the formation of meristematic cells during root primordium formation (Imin et al., 2007; Passarinho et al., 2008).

### WUSCHEL-RELATED HOMEOBOX (WOX) Family TFs

The expression of *WUSCHEL*-*RELATED HOMEOBOX* (*WOX*) family TF genes characterizes the early derivatives of AR founder cells in Arabidopsis (Sarkar et al., 2007; Liu et al., 2014). The auxin-inducible *WOX5* specifically expresses in the QC in Arabidopsis (Sarkar et al., 2007) and plays a pivotal role in the RAM formation in leaf cultures of *Medicago truncatula* (Chen et al., 2009). WOX5 can maintain the maximum auxin level at the root tip by affecting auxin distribution in Arabidopsis (Gonzali et al., 2005; Ditengou et al., 2008; Ding and Friml, 2010). In *A. thaliana* leaf explants, *WOX11* and *WOX12* were also shown to be involved in *de novo* root organogenesis (Liu et al., 2014). *WOX11* acts redundantly with *WOX12* to upregulate *LBD16* and *LBD29*, resulting in the formation of root founder cells. Mutations in the WOX-binding elements caused reduced expression of *LBD16* during AR generation, indicating that *WOX11/12* directly activate *LBD16* by binding to the elements of the LBD16 promoter (Xu, 2018Figure 2). Furthermore, WOX11/12 activates WOX5/7 transcription by directly binding to their promoters. Mutations in WOX5/7 result in the defect in primordium formation. These results indicate that the molecular process from WOX11/12 to WOX5/7 plays a critical role in root primordium initiation (Hu and Xu, 2016). In poplar, WOX family genes are also determined to be involved in AR formation. For example, Xu et al. (2015) demonstrated that overexpressing either *PeWOX11a* or *PeWOX11b* increased the AR number on the cuttings of poplar. A recent study cloned a *Larix kaempferi* gene *LkWOX4* and overexpressed it in poplar, and the result showed that *LkWOX4* overexpression significantly increased AR numbers and decreased AR lengths (Wang et al., 2020), imply that the common role of *WOX* members in modulating AR formation in different plant species.

### Co-expression of *AP2/ERPB2* and *WOX* TF Family Genes During RAM Formation

AP2/ERPB2 domain TFs genes *BBM*, *PLT2*, and *WOX5* are highly expressed in the root formation cultures of *Medicago truncatula*, reflecting the auxin-induced enrichment of these genes within root stem cells (Holmes et al., 2010). In *Arabidopsis*, the *WOX5*, *SCR*, *SHR*, *PLT1*, and *PLT2* genes are all involved in the control of the formation of RAM and QC (Aida et al., 2004; Xu et al., 2006). The *Medicago truncatula MtWOX5*, *MtPLT1*, *MtPLT2*, and *MtBBM1* genes are also shown to regulate the formation of RAM (Imin et al., 2007). The *Populus trichocarpa* gene *PtAIL1*, which is an ortholog of *Arabidopsis AtANT*, was shown to promote the formation of root primordia. Rigal et al. (2012) demonstrated that, during AR formation in poplar cuttings, the *ANT* and *AIL* genes were activated by auxin and cytokinin.

### Other TF Family Genes

The TF families zinc finger, WRKY, NAC, and bZIP have also been confirmed to be involved in RAM formation. For example, an auxin-inducible zinc finger TF gene *LATERAL ROOT PRIMORDIA1* (*LRP1*) (Smith and Fedoroff, 1995; Holmes et al., 2010), a WRKY family member *WRKY75* (Devaiah et al., 2007), a NAC family member *NAC1* (petunia *NAM* and Arabidopsis *ATAF1, ATAF2*, and *CUC2*) (Chen et al., 2016), and a bZIP family member bZIP53 (Zhang et al., 2020) are involved in AR primordium formation and AR tip emergence. The auxin-inducible MYB-DOMAIN PROTEIN77 (MYB77) TF gene may be involved in AR initiation by interacting with ARFs and enhancing the expression of auxin-responsive genes (Shin et al., 2007).

### Other Genes Involved in RAM Formation

In Arabidopsis, *ROOT INITIATION DEFECTIVE2* (*RID2*) encodes a nuclear methyltransferase-like protein that likely contributes to the nucleolar activity of pre-rRNA processing via methylation, thereby promoting cell proliferation and consequently maintaining cell proliferation competence during dedifferentiation (Ohbayashi et al., 2011). *ROOT PRIMORDIUM DEFECTIVE1* (*RPD1*), *ROOT GROWTH DEFECTIVE1* (*RGD1*), *RGD2*, and *RGD3* play roles in the maintenance of active cell proliferation in AR primordia (Konishi and Sugiyama, 2003, 2006). *ROOT CLAVATA1*-*HOMOLOGUE1* (*RCH1*) and *RCH2* encode the leucine-rich repeat receptor-like kinases, which are detected to specifically express in RAM cells (Dello Ioio et al., 2007). *MICOTUBULE ORGANIZATION1* (*MOR1*) encodes a microtubule-associated protein, which is involved in auxin-responsive root meristem initiation (Konishi and Sugiyama, 2003). The gamma-glutamylcysteine synthetase-coding gene *ROOT MERISTEMLESS1* (*RML1*) mediates meristem initiation and the maintenance of cell division during AR formation in *Arabidopsis* and *Medicago* (Vernoux et al., 2000b; Holmes et al., 2010). Gamma-glutamylcysteine synthetase acts as the first enzyme in glutathione biosynthesis, implying that glutathione pathway is involved in the regulation RAM. The CYCLIN-DEPENDENT KINASE2 (CDC2) acts as a developmental switch between mitotic cell division and post-mitotic cell differentiation and maintains the proliferation competence. In *Pinus contorta, PcCDC2* is an auxin- and cytokinin-inducible gene, which functions as the role of CDC2 in cell division competence during auxin-induced AR formation in cuttings (Anders et al., 2001). The *Olea europaea* alternative oxidase gene *OeAOX2* is highly expressed in AR primordia (Peixe et al., 2007) and its activity is highly induced during adventitious rooting in cuttings (Macedo et al., 2009). The heme oxygenase-1 gene *HO-1* is involved in adventitious rooting in cucumber via the regulation of the HO-1-mediated target genes, such as *DNAJ-like* and the gene encoding calcium-dependent protein kinase (*CDPK*) (Lin et al., 2012).

## Molecular Bases of Cell Wall Modification During the Emergence of AR Tips

The emergence of AR primordium through cortex and epidermis cells requires cell wall degradation and reconstruction (Steffens and Rasmussen, 2016). The EXTENSIN (EXT) functions as a wound healing protein and might limit the emergence of AR tips (Chen et al., 2016). The accumulation of EXT at the wounded site strengthens cell walls during wound healing in Arabidopsis (Merkouropoulos and Shirsat, 2003). Wounding induced the expression of *EXT* during rooting in *Vitis vinifera* stem cuttings (Thomas et al., 2003). In the leaf explants of Arabidopsis, wounding induces the expression of *NAC1*, which then enhances the expression of *Cys-ENDOPEPTIDASE* (*CEP*). The elevated CEP activity promotes the degradation of EXT. Thus, NAC1-CEP pathway promotes the emergence of AR tips via antagonizing EXT-mediated wound healing during AR emergence (Chen et al., 2016).

Many other genes have been shown to participate in the cell wall remodeling during AR emergence (Rigal et al., 2012). *HYP-RICH GLYCOPROTEIN* (*HRGP*), a cell wall protein family gene, is specifically activated during AR initiation in tobacco cuttings (Lund et al., 1997). *ROOT HAIR DEFECTIVE3* (*RHD3*), a large GTP-binding protein-coding gene, is mediated in cell wall biosynthesis and actin organization, both of which are essential for cell expansion during AR formation in Arabidopsis and *Populus* (Hu et al., 2003; Xu et al., 2012). Expansins are responsible for the acid-induced loosening of cellulose and hemicellulose and are induced in hypocotyl bases during the early stages of AR induction in the cuttings of *Pinus taeda* (Hutchison et al., 1999). *AtPME3* encodes a basic pectin methylesterase 3PME that plays a role in AR emergence in *Arabidopsis*. The absence of this protein leads to changes in the degree of methylesterification of galacturonic acids in cell wall components (Guenin et al., 2011). *OeAOX* promotes adventitious rooting in the cuttings of *Olea europaea* by linking phenylpropanoid metabolism and lignin metabolism (Macedo et al., 2012).

## Molecular Bases for the Crosstalk of Plant Hormone Pathways During Adventitious Rooting

### Ethylene-Auxin Crosstalk During AR Formation

Many studies have shown that AR formation also involves ethylene signaling pathway and the crosstalk with auxin (Negi et al., 2010; Vidoz et al., 2010; Figure 2). In Arabidopsis, auxin can enhance ethylene level by inducing the expression of several ACC synthase genes (Stepanova et al., 2005; Negi et al., 2008), thereby increasing ethylene levels and activating the expression of *ARF19* via the two tissue-specific anthranilate synthases (Ruzicka et al., 2007; Stepanova et al., 2007; Swarup et al., 2007). Ethylene can promote IAA synthesis via inducing the expression of *WEI2/ASA1* and *WEI7/ASB1* genes (Ruzicka et al., 2007; Stepanova et al., 2007; Swarup et al., 2007) and promotes polar auxin transport via regulating the expression of the IAA-efflux carriers PIN1, PIN2, and PIN4, and the IAA-influx carriers AUX1 and LAX3 (Swarup et al., 2007; Negi et al., 2008, 2010). Both auxin and ethylene directly activate the expression *ARF19* and *ARF7* (Li et al., 2006). Therefore, ARF19 and ARF7 act as mediators of crosstalk between auxin and ethylene signaling pathways. The WEI2/ASA1 and WEI7/ASB1 proteins introduce into a feedback control between auxin and ethylene (Ivanchenko et al., 2008). For example, during flood-induced stem AR formation in tomato, WEI7 promotes the Trp-dependent auxin biosynthesis and triggers additional ethylene synthesis by enhancing the expression of two ACC synthase genes, *LeACS3* and *LeACS7*, and DIAGEOTROPICA (DGT) is involved in this pathway (Vidoz et al., 2010). DGT has been shown to regulate auxin transport by affecting PIN localization (Ivanchenko et al., 2015). During etiolation-induced AR formation in Arabidopsis, ethylene initiates the *ETHYLENE-INSENSITIVE2* (*EIN2*)-*EIN3-like* (*EIL1*) transcriptional cascade resulting in the inhibition of WEI2 (ASA1) and WEI7 (ASB) and YUCCA6 and consequently, reduces IAA level and AR formation (Veloccia et al., 2016).

Besides, many studies have shown that AP2/ERF family genes are also involved in AR formation. For example, the *PtaERF003* gene, a member of the AP2/ERF family, promotes adventitious rooting in poplar cuttings through auxin signaling pathway (Trupiano et al., 2013). In a hybrid poplar clone, downregulated expression of *PtaERF003* results in low auxin accumulation in competent cells and consequently, reduced AR formation (Trupiano et al., 2013). The S-adenosylmethionine synthase (SAMS) catalyzes the production of S-adenosylmethionine and is involved in ethylene synthesis. Brinker et al. (2004) found that the *P. contorta* SAMS-coding gene *PcSAMS1* is involved in AR meristem formation.

### Cytokinin-Auxin Crosstalk During AR Formation

**FIGURE 4:**
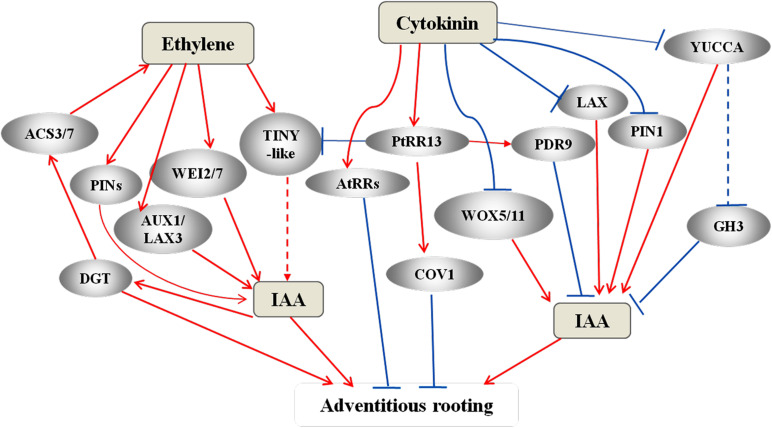
A complex network of IAA, ethylene, and cytokinin that has been proposed to regulate AR formation. In the AR models of Arabidopsis and flooded tomato, auxin triggers ethylene synthesis through the expression of *DIAGEOTROPICA* (*DGT*) and ACC synthesis genes *ACS3* and *ACS7* (Stepanova et al., 2005; Negi et al., 2008; Vidoz et al., 2010). Ethylene promote IAA synthesis via activating of WEI2 and WEI7 (Ruzicka et al., 2007; Stepanova et al., 2007; Swarup et al., 2007) and promotes polar auxin transport via activating PINs and AUX1 and LAX3 (Swarup et al., 2007; Negi et al., 2008, 2010). Cytokinin signaling activates RRs to repress AR in Arabidopsis (Argyros et al., 2008). In the AR model of poplar cuttings, the active PtRR13 repress AR formation by promoting the expression of *COV1* and *PDR9* and also by inhibiting the expression of TINY-like transcription factors (Ramirez-Carvajal et al., 2009). TINY is an activator of ethylene signaling (Sun et al., 2008) and a possible intersection point between ethylene and cytokinin signaling. Cytokinin also regulates auxin homeostasis and accumulation by negatively affecting the auxin carriers PIN1 and LAX3, and the *WOX* and *YUCCA* genes, thereby regulating adventitious rooting through these pathways, as well. Red arrows represent positive regulation, and blue bars represent negative regulation. The dotted lines or arrows represent the pathways need further identified.

Cytokinin represses the differentiation of primordia and AR formation in cucumber hypocotyls and in *Populus tremula* cuttings (Kuroha et al., 2002; Ramirez-Carvajal et al., 2009). Cytokinin negatively regulates auxin by inducing AUX/IAA proteins and downregulates *PIN* expression (Moubayidin et al., 2009; Su et al., 2011). Furthermore, in *Arabidopsis*, cytokinin inhibits *PIN1* and *LAX3* expression and further blocks auxin flow. It also limits the expression of *WOX5* and *YUCCA6* at the distal tip (Rovere et al., 2013). The type-B cytokinin response regulators (RRs) are cytokinin-responsive transcriptional activators, acting as histidine kinases (HKs) (Nishimura et al., 2004). *Arabidopsis* mutations with defect in type-B RRs are characterized by insensitivity to cytokinin and instinctive formation of ARs in hypocotyls (Argyros et al., 2008). In *P. tremula* cuttings, PtRR13 transcriptionally regulates downstream cytokinin signaling to repress adventitious rooting (Ramirez-Carvajal et al., 2009). Moreover, PtRR13 promotes the expression of *CONTINUOUS VASCULAR RING1* (*COV1*), which encodes a negative regulator of vascularization, and *PLEIOTROPIC DRUG RESISTANCE TRANSPORTER9* (*PDR9*), which encodes an auxin efflux pump, further affecting the vascular tissue formation during adventitious rooting. Thus *PDR9* acts as a mediator of crosstalk between cytokinin and auxin signaling (Figure 4). In addition, PtRR13 represses adventitious rooting by inhibiting the expression of two ethylene-inducible TINY-like TFs belonging to the DEHYDRATION-RESPONSIVE ELEMENT-BINDING (DREB) protein subfamily of AP2/ERF TFs. TINY is a activator of ethylene signaling (Sun et al., 2008) and a possible intersection point between ethylene and cytokinin signaling (Ramirez-Carvajal et al., 2009).

During adventitious rooting in cultures of *Medicago truncatula*, cytokinin inhibits the *PLT1, PLT2*, and *BBM1* expression and induces the expression of *ANT, AIL1*, and the TFs *STM* and *WOX4* (Imin et al., 2007). WOX11 functions as an integrator of auxin and cytokinin signaling by directly repressing the expression of *RR2* and is involved in the cytokinin-regulated development of ARs (Zhao et al., 2009; Kitomi et al., 2011).

### Jasmonic Acid-Auxin Crosstalk During AR Formation

Jasmonic acid (JA), a stress-related hormone, inhibits adventitious rooting and works downstream of the IAA pathway (Gutierrez et al., 2012) through the GH3 proteins in Arabidopsis (Staswick et al., 2005; Staswick, 2009). GH3s catalyze the conjugation of IAA with amino acids and JA with amino acids, thereby reducing free IAA level and controlling JA homeostasis. Among them, GH3.11, also known as JASMONIC ACID RESISTANT1 (JAR1), is shown to suppress adventitious rooting in *Arabidopsis* hypocotyls. JAR1 conjugates JA to Ile to produce JA-Ile, an active form of JA, which further activates the JA receptor CORONATINE INSENSITIVE1 (COI1) (Fonseca et al., 2009; Figure 1) and promotes the interaction of COI1 with the co-receptor JAZ, which also is a transcriptional repressor of JA-responsive genes (Thines et al., 2007). GH3.3, GH3.5, and GH3.6 catalyze the production of JA-Asp, JA-Met, and JA-Trp, which are inactive forms of JA. Thus, these three GH3s have opposite effect to that of GH3.11 during adventitious rooting (Gutierrez et al., 2012). A study reveals a crosstalk between auxin and JA pathways that fine-tunes AR initiation in *Arabidopsis* hypocotyls (Gutierrez et al., 2012). Auxin affects JA homeostasis by upregulating the expression of *GH3.3*, *GH3.5*, and *GH3.6*, resulting in an increase in conjugational JA and a decline in the free JA. Auxin also induces the expression of JA biosynthetic genes in *Arabidopsis* (Paponov et al., 2008). Furthermore, during adventitious rooting in *Arabidopsis*, another linkage between IAA and JA involves the interaction between the auxin-responsive genes *ARF6, ARF8*, and *ARF17* and their target genes *GH3.3, GH3.5*, and *GH3.6*. Recently, Lakehal et al. (2019a) proposed TIR1/AFB2-Aux/IAA-dependent auxin signaling, in which TRANSPORT INHIBITOR1 (TIR1) and AUXIN-SIGNALLING F-BOX (AFB2) proteins interact with IAA6, IAA9, and/or IAA17 to control JA homeostasis and AR initiation in Arabidopsis (Figure 4). Another feedback circuit between IAA and JA is mediated by DIOXYGENASE FOR AUXIN OXIDATION (DAO1) and COI1-dependent JA signaling. AtDAO1 catalyzes the production of 2-oxindole-3-acetic acid (oxIAA), thereby reducing free IAA level and AR initiation in Arabidopsis. The expression of *DAO1* is induced by COI1-dependent JA signaling. DAO1 controls IAA level by catalyzing the conversion of IAA to oxIAA (Lakehal et al., 2019b).

However, MeJA (methyl jasmonate), another form of JA, is shown to promote adventitious rooting from thin cell layers of *Nicotiana tabacum* (Fattorini et al., 2009). MeJA increases the endogenous IAA and JA levels in *Arabidopsis* (Paponov et al., 2008). MeJA may maintain auxin homeostasis during adventitious rooting through regulating the coordination of PIN, IAA-amino acid hydrolase, and IAA-amido synthetase (Chen et al., 2007; Fattorini et al., 2009). For example, MeJA enhances the activity of PIN and IAA-amino acid hydrolase 6. Considering positive effects of MeJA also on IAA levels and the expression of ASA1 in Arabidopsis thin cell layers (Fattorini et al., 2017, 2018), further the early JA accumulation in the stem base of petunia cuttings (Ahkami et al., 2009) and furthermore the finding that reduced expression of the rate-limiting enzyme ALLENE OXIDE CYCLASE (PhAOC) in petunia inhibited wound-induced JA accumulation and AR formation (Lischweski et al., 2015), Druege et al. (2019) proposed that early wound-induced JA accumulation in cuttings stimulates AR induction, possibly via enhanced IAA accumulation. According to this theory, up-regulation of ASA1, IAA accumulation and intensity of AR formation in Arabidopsis leaf explants was related to early JA accumulation and dependent on JA signaling (Zhang et al., 2019).

### Gibberellin (GA)-Auxin Crosstalk During AR Formation

The GA biosynthetic gene *GA20ox1-OE* plays an important role in GA homeostasis (Gallego-Giraldo et al., 2008). *GA-INSENSITIVE DWARF1* (*GID1*) is a GA receptor gene (Ueguchi-Tanaka et al., 2005). Both hybrid aspens and *Arabidopsis* mutation lines with overexpression of *AtGA20ox1*, *PttGID1.1*, or *PttGID1.3* genes display defects in AR formation, indicating that GA inhibits ARs by perturbing GA synthesis or signaling and polar auxin transport (Mauriat et al., 2014). A recent study cloned a histone deacetylase (*HD2*) gene *PtHDT902* from *Populus trichocarpa*. Overexpressing *PtHDT902* in Arabidopsis and poplar enhanced the expression of GA biosynthetic genes and inhibited AR formation in poplar (Ma et al., 2020), suggesting that HD2 promotes GA biosynthesis and thus suppresses AR formation.

## Perspectives

In recent years, there have been significant advances in our understanding of the contributions of hormone signaling and gene networks to the regulation of the formation of ARs. An increasing number of genes involved in adventitious rooting and their interactions have been characterized and identified. The diversity of AR types and the complexity of the gene regulation networks pose a challenge to researchers. However, the molecular networks and mechanisms underlying the development of ARs need to be further clarified. At the transcriptional level, it is necessary to ascertain the key genes and gene expression profiles that regulate the different AR types and different stages of AR development. At the post-transcriptional level, the key proteins and protein profiles, as well as their regulation mechanisms during AR formation need to be explored. Another interesting issue is about the inherent differences between the easy-to-root and difficult-to-root plants at the molecular level. Such knowledge should ultimately lead to a better understanding the mechanisms underlying plant cell differentiation and AR development. In practice, we need to exploit the potential of ARs for improving the crop tolerances to various environmental stresses. Rapidly expanding knowledge of plant genomes and proteomes and new technologies will most likely shed more light on these questions in the future.

## Author Contributions

The author confirms being the sole contributor of this work and has approved it for publication.

## Conflict of Interest

The author declares that the research was conducted in the absence of any commercial or financial relationships that could be construed as a potential conflict of interest.
